# Predicting the spread of SARS-CoV-2 variants: An artificial intelligence enabled early detection

**DOI:** 10.1093/pnasnexus/pgad424

**Published:** 2024-01-02

**Authors:** Retsef Levi, El Ghali Zerhouni, Shoshy Altuvia

**Affiliations:** Sloan School of Management, Massachusetts Institute of Technology, Cambridge, MA 02139, USA; Operations Research Center, Massachusetts Institute of Technology, Cambridge, MA 02139, USA; Department of Microbiology and Molecular Genetics, The Hebrew University-Hadassah Medical School, Jerusalem, 9112102, Israel

**Keywords:** SARS-CoV-2 variants, computational biology, machine learning, genetics, epidemiology

## Abstract

During more than 3 years since its emergence, SARS-CoV-2 has shown great ability to mutate rapidly into diverse variants, some of which turned out to be very infectious and have spread throughout the world causing waves of infections. At this point, many countries have already experienced up to six waves of infections. Extensive academic work has focused on the development of models to predict the pandemic trajectory based on epidemiological data, but none has focused on predicting variant-specific spread. Moreover, important scientific literature analyzes the genetic evolution of SARS-CoV-2 variants and how it might functionally affect their infectivity. However, genetic attributes have not yet been incorporated into existing epidemiological modeling that aims to capture infection trajectory. Thus, this study leverages variant-specific genetic characteristics together with epidemiological information to systematically predict the future spread trajectory of newly detected variants. The study describes the analysis of 9.0 million SARS-CoV-2 genetic sequences in 30 countries and identifies temporal characteristic patterns of SARS-CoV-2 variants that caused significant infection waves. Using this descriptive analysis, a machine-learning-enabled risk assessment model has been developed to predict, as early as 1 week after their first detection, which variants are likely to constitute the new wave of infections in the following 3 months. The model’s out-of-sample area under the curve (AUC) is 86.3% for predictions after 1 week and 90.8% for predictions after 2 weeks. The methodology described in this paper could contribute more broadly to the development of improved predictive models for variants of other infectious viruses.

**Significance Statement**

The surveillance of SARS-CoV-2 variants is a priority for the Centers for Disease Control and Prevention and World Health Organization to inform pandemic preparedness. However, even after most countries experienced six waves of infections, it is still challenging to identify in a timely manner, the very small fraction of variants that will cause a new wave. Based on 9.0 million SARS-CoV-2 genetic sequences in 30 countries, the study develops an innovative approach to predict the future spread of new variants. It starts with the quantitative characterization of temporal patterns of infectious variants, and their respective genetic attributes. This is then used to design machine-learning-based algorithms with strong predictive power to detect infectious variants in each country they appear as soon as 1 or 2 weeks after their detection.

## Introduction

For over 3 years, the SARS-CoV-2 virus has spread throughout the world, leading to multiple waves of infections, and imposing a tremendous health, economic, and social toll on most countries. Furthermore, the virus has shown great ability to rapidly mutate into more infectious variants, including ones with the ability to escape the available vaccines.

Since the original wild-type virus that emerged from Wuhan, China, and throughout 2023 August 26, there have been >2,924 documented variants that were sequenced worldwide (1). The US Centers for Disease Control and Prevention (CDC) and World Health Organization (WHO) classify these variants into three main categories, including Variants Being Monitored, Variants of Interest, and Variants of Concern. A variant is classified as of “interest” when it is associated with community transmission in addition to genetic changes, while a variant of “concern” when it shows evidence of increased transmissibility, virulence, or is associated with decreased effectiveness of the public health measures in place (2, 3). However, this classification remains somewhat qualitative with no precise or consistent metrics and thus allowing little underlying predictive power.

For instance, the UK Health Security Agency classified the variant Alpha that was first detected in the United Kingdom on November 1, as a variant of concern only on 2020 December 18 (4), at which point in time, it has already spread in the country and accounted for half of the 191,000 new cases (1, 5). Similarly, the same agency did not classify the variant AY.4 as of concern (6), as late as of 2021 August 16, despite the fact that it accounted for >70% of the SARS-CoV-2 sequences collected in the United Kingdom (1, 5). At the international level, the WHO classified the Delta (B.1.617.2) variant as of concern only on 2021 May 11 (3), whereas it has been first detected in India in December 2020, and then spread to the United Kingdom and United States in April 2021. Interestingly, the spread trajectory of variants varies not only across variants but also for the same variant across different countries. For example, the variant B.1.177 led to >3,017 reported infection cases per million in the United Kingdom, while it caused only 14 new cases per million in the United States 3 months after its detection in these respective countries.

As part of their activities to surveil and assess circulating variants, both the US government and the WHO emphasize the scaling of genetic sequencing capabilities and improved predictive models to better surveil future variants as two critical enablers of improving preparedness to future pandemics (7, 8). Specifically, the American *National COVID-19 Preparedness Plan* fosters the need to prepare for new variants. As part of this plan, the CDC scaled its genetic sequencing capabilities with the *National SARS-CoV-2 Strain Surveillance* program to reliably detect variants that account for as little as 0.1% of all cases circulating in the United States (9). These efforts emphasize the need to rapidly identify infectious variants, allowing the government to respond quickly (10).

Aligned with these priorities, multiple research groups around the world have been closely monitoring the evolutionary epidemiology of SARS-CoV-2. This led to the development of appropriate epidemiologic models for SARS-CoV-2 that account for the effect of its genetic evolution on the pandemic trajectory (11), as well as yielded important empirical observations regarding its adaptive evolution with immune-escape mutations, continuous diversification, and regional specification (12). Specifically, there has been a great emphasis on tracking the emergence of new variants, or even new lineages, and analyzing their respective mutations to explain the resurgence of SARS-CoV-2 infection waves (13, 14). The hope is that increasing genomic surveillance capabilities would provide the basis to develop early warning capabilities in assessing whether a newly detected variant would become widely circulating in a specific geographic area (15–17). Ideally, this desired capability should rely on predictive capabilities to identify as early as possible those variants that are more likely to be infectious and lead to a large number of infections. Currently, the CDC relies on relatively short-term predictions of variants, specifically predicting their relative proportions 2 to 3 weeks ahead (9). For longer term predictions, although multiple forecasting models have been developed during the pandemic (18, 19), the substantial volume of genetic data collected on the virus thus far has yet to be used for variant and country-specific predictions.

This study aims to develop a data-driven approach to assess the spread risk associated with different SARS-CoV-2 variants, and create a machine-learning-enabled framework to allow a more consistent classification of new variants and early prediction of their likely long-term spread trajectory as early as 1 week after their detection. The analysis in the paper is based on data from 30 countries, leveraging multiple sources. These include the commonly used Pango lineage nomenclature of SARS-CoV-2 variants (20), >9.0 million SARS-CoV-2 genetic sequence data collected by the Global Initiative on Sharing Avian Influenza Data (GISAID) (1), as well as reported nonpharmaceutical interventions, SARS-CoV-2 infections, and vaccination rates per country (5). The paper first provides a descriptive analysis of the temporal patterns within and between SARS-CoV-2 infection waves, and specifically, their relation to circulating variants and their respective genetic diversity. Subsequently, the paper develops a machine-learning-enabled predictive model for early detection of variants’ spread in each country.

## Results

### Definition of SARS-CoV-2 infection waves and their analysis

This study focuses on 30 countries that have reported the highest number of SARS-CoV-2 genetic sequences throughout 2022 March 19. Among these countries, the United States has the highest number with 2.9 million reported sequences, and Luxembourg has the smallest number with 25,000 reported sequences. Overall, these 30 countries capture 9.0 million of total 9.5 million genetic sequences reported in GISAID (1) since the beginning of the pandemic. The Pango lineage is commonly used by the WHO, CDC, and other public health agencies in Europe to classify variants and their evolution (20). Following this nomenclature, by 2022 March 19, there are 1,151 distinct variants consistently identified in the countries under study, with a median of 72 (25% quantile [q1]: 57, 75% quantile [q3]: 121) detected variants per country since the beginning of the pandemic (see Table S1 for the count of variants in every country).

Since there is no standardized definition of a “wave” of SARS-CoV-2 infections, this paper adapts and integrates existing approaches. Specifically, the newly proposed methodology identifies the beginning of the first wave of SARS-CoV-2 infections in a given country when the first case is detected. The wave ends when the 7-day average of new infections goes below one-tenth (0.1) of the peak of the wave. Then, each subsequent wave starts whenever the last 7-day average count of new infections goes above one-tenth of the maximal peak in the previous wave or 100 new infections per million citizens. The wave ends based on the same criteria as before. This methodology combines elements from incidence-based methods that detect infection waves by comparing the incidence rate of cases to a preset threshold (21, 22), as well as change point detection methods which enable to detect waves that start meanwhile a previous wave is still decaying (23–25).

When these methods are applied independently, they either miss waves or are too sensitive to identify “additional” waves (see Fig. S4). In contrast, the paper’s methodology described above matches the CDC and WHO wave definitions. The latter are based on the variant that drives infections, such as the wave driven by the Beta (B.1.351) variant, or the following wave driven by the Delta (B.1.617.2) variant (26–28). Applying the paper’s methodology, a total of 144 waves of SARS-CoV-2 infections were identified in the 30 countries under study throughout 2022 March 19, out of which 115 waves have already ended by then. Moreover, the median number of waves per country is 5 (q1: 4, q3: 5), and the median length of a wave is 119 days (q1: 95, q3: 154).

The subsequent analyses focus on the waves that ended by 2022 March 19 and identify patterns in the circulating variants during the respective waves. The median number of distinct circulating variants detected within the duration of a given wave is 102 (q1: 59, q3: 166). However, each wave in a specific country has what is defined as a first *dominant variant*, which is the variant with the largest number of documented SARS-CoV-2 genetic sequences within the duration of the respective wave. Similarly, in each wave, the second (resp. third) dominant variant corresponds to the variant with the second (resp. third) highest number of reported genetic sequences within the duration of the wave.

**Fig. 1.**
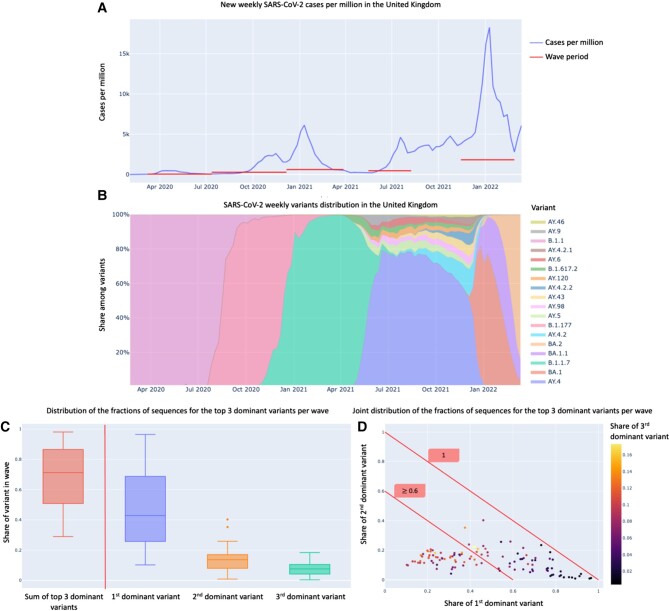
Progression of SARS-CoV-2 variants’ distribution. A) Weekly SARS-CoV-2 cases and B) weekly variants’ distribution in the United Kingdom based on the reported sequences in GISAID. C) Distribution of the fractions of sequences for the first, second, and third dominant variant sequences, and their sum, among all the sequences reported in every wave. D) Joint distribution of the fractions of sequences for the first, second, and third dominant variant sequences among all the sequences reported in every wave.

Figure 1A and B present, respectively, the SARS-CoV-2 cases over time in the United Kingdom, and based on the sequencing data, the estimated relative proportion of cases is associated with each variant over time. It illustrates that each new wave of infection is typically associated with a specific new set of dominant variants. In particular, the four waves starting, respectively, in April 2020, October 2020, January 2021, and July 2021 were driven, by variants B.1.1, B.1.177, Alpha (B.1.1.7), and Delta (B.1.617.2, AY.4, and AY.4.2). Furthermore, the fifth wave, starting in November 2021, has been driven by the Omicron variants (BA.1, BA.1.1, and BA.2).

These are only 9 out of the 436 variants identified in the United Kingdom throughout 2022 March 19. Figure 1C extends this analysis to the 30 countries under study and plots the respective distributions of the relative shares of the top three dominant variants across all identified waves. Figure 1D plots the joint distributions of the relative shares of infections of first dominant and second dominant variants across all waves. The distributions in Fig. 1C show that the top three dominant variants are typically responsible for most of the cases during the respective wave with a median of 71.3% cumulative share (q1: 50.8%, q3: 86.5%) across all waves. Specifically, for the first, second, and third dominant variants in each wave, the median shares are 42.9% (q1: 25.8%, q3: 68.8%), 13.7% (q1: 8.0%, q3: 17.1%), and 7.6% (q1: 4.2%, q3: 10.6%), respectively. Figure 1D also suggests that in most waves, the first dominant variant has a significantly higher share of infections than the second dominant variant, and that in more than half of the waves (69 out of 115), the top two most dominant variants account for over 60% of the infections in the wave. Furthermore, in 104 out of the 115 waves, the first dominant variant was neither first dominant nor second dominant in the preceding wave in that country. In seven additional waves, the dominant variant of the wave was only second dominant in the previous wave. Finally, 5 out of the 9 waves, where the dominant variant in the respective wave was also dominant in the previous wave, had a relative share smaller than 35% in the previous wave compared with >80% in the current wave (see Table S2 for details on these 9 waves).

### Variants’ genetic distance between waves

Each new variant can be characterized by several mutations in the various proteins of the virus with respect to the hCoV-19/Wuhan/WIV04/2019 (GISAID reference: EPI-ISL-402124) detected in Wuhan in early January 2020. This variant was chosen as the reference genome sequence (1, 29). Mutations typically occur in viral proteins, including the spike that binds to receptors on human cells (30), the structural nucleocapsid (N), the membrane (M), and envelope (E) involved in the virus building process (31), and the nonstructural proteins (NSPs), responsible for the virus replication (32). These proteins’ amino acid sequences are of different sizes, with 1,273, 75, 22, 419, and 7,176 amino acids for the spike, E, M, N, and NSP, respectively, and have different mutation rates (33).

The subsequent analysis considers all possible mutations in a genetic sequence, including base substitutions, deletions, and insertions (34). The reported numbers of these mutations in each of the virus’ proteins across all variants indicate that the highest number of mutations occur in spike, N, and NSP proteins with median number of mutations per variant in each country of 10 (q1: 2, q3: 12), 3 (q1: 2, q3: 4), and 14 (q1: 6, q3: 19), respectively (see Fig. S1 for the distribution of mutations count in all proteins). Additionally, mutation rates, corresponding to the number of mutations per variant normalized by the length of the protein’s amino acid sequence, are highest for spike and N proteins with a median of 0.78% (q1: 0.16%, q3: 0.94%) for spike and 0.71% (q1: 0.47%, q3: 0.95%) for N. The NSP has a lower mutation rate with a median of 0.20% (q1: 0.08%, q3: 0.26%).

The mutations of each variant with respect to the baseline Wuhan sequence enable to measure the genetic distance from one variant of the virus to another. The study uses the *Jaccard* distance metric (35) and the variant-specific set of mutations compared with the baseline virus strain to construct a novel distance metric between different variants. For each pair of variants, the distance is calculated based on their respective specific set of mutations, as the ratio between the total number of mutations that are unique to exactly one of the variants, and the total number of mutations of the two variants, common or unique. It is readily verified that the Jaccard distance receives any value from 0 to 1, where 0 corresponds to a situation where the two sets of variant-specific mutations are in fact identical and 1 corresponds to a situation when the two sets have no common mutation whatsoever.

Notably, a more traditional method to compute the genetic distance would be the phylogenetic distance (36). This metric considers the direct comparison of the entire genetic sequence, or part of it, and not merely the mutations with respect to a baseline variant. As a result, the phylogenetic distance is quite effective in capturing longitude genetic evolution, but unfortunately, is less useful in capturing differences between variants in the shorter term. This study focuses mostly on short-term changes over the course of several weeks or months. Thus, the phylogenetic distance displays barely detectable variations between pairs of variants, in contrast to the Jaccard distance that enables to better capture these short-term changes. Analyses described in SI Appendix illustrate in more detail the respective ability of the Jaccard and phylogenetic distance metrics, to distinguish between variants that dominate different waves, as well as between variants that ultimately become infectious vs. not. These analyses further motivate the use of the Jaccard distance in this study.

The subsequent analysis attempts to leverage the Jaccard distance to better understand how the mutations of each variant change over time from one wave of infections to the next and identify potential predictors of the spread trajectory. Specifically, the analysis describes how distinct the mutations of the first dominant variant in each wave are, from all the circulating variants and their respective mutations during the previous wave. This metric is called *interwave* distance and computed as the weighted average distance between the dominant variant of a given “current” wave and the variants reported during the previous wave. The pairwise distance is weighted by the relative share the respective variant in the previous wave (see Materials and methods for precise mathematical definitions).

The results of this analysis indicate that typically the first dominant variant in each wave has relatively very distinct mutations when compared with the variants circulating in the previous wave, with a median interwave distance of 0.90 (q1: 0.78, q3: 0.95). In fact, 63.9% (q1: 47.7%, q3: 86.1%) of the mutations defining a new dominant variant do not seem to appear in circulating variants within the prior wave, while 86.9% (q1: 74.2%, q3: 92.4%) of the mutations in the latter group of variants are not inherited by new dominant variants. Additionally, when compared with only the first dominant in the previous wave, each new dominant variant has a Jaccard distance of 0.95 (q1: 0.88, q3: 0.96), with 77.7% (q1: 52.7%, q3: 92.8%) of its mutations being new, and 93.1% (q1: 85.7%, q3: 93.8%) of the mutations in the previous dominant variant not inherited by the new one. This supports the hypothesis that variants that drive new waves of infections need a significantly different mutational profile to overcome the partial immunity in a population created by previous waves (37, 38).

### Variants’ genetic diversity within waves

In addition to comparing the genetic attributes of variants across successive waves, it is also important to characterize their mutational diversity within each wave. To do so, the study introduces two metrics to characterize the diversity of variants during a specified period of time.

The two metrics capture mutational diversity and consider the number of circulating variants, the genetic distances between them, and their relative shares during the specified period of time. These features are motivated by the effects of variants’ diversity on the likelihood that a new variant acquires phenotypic characteristics such as transmissibility or immune evasion (39). The first metric, called hereafter *variants entropy*, is inspired by the application of the thermodynamic concept of entropy in ecological systems to compare between states of low and high entropy, corresponding, respectively, to whether there are only a few or many cocirculating variants (40–42). Specifically, variants’ entropy during any predefined period is calculated as the negative of the product between a variant’s relative share of infections and the logarithm of this ratio, summed over all the variants circulating during the period of interest.

A complementary measure of variants’ diversity, called hereafter *variants’ heterogeneity*, captures both the distributions, i.e. number of variants and their respective fraction of infections, as well as the genetic distances between the circulating variants. This metric is calculated, during the period of interest, as the weighted average of Jaccard distances across all pairs of circulating variants, with weights being the product of the shares of reported genetic sequences for the respective pair of variants (see Materials and methods for precise mathematical definition). Meanwhile, variants’ entropy primarily measures the number of distinct variants and the respective number of infections each causes, variants’ heterogeneity captures how genetically distinct they are. High heterogeneity values would correspond to a state where infections are caused by multiple variants with relatively distinct mutations, whereas low heterogeneity values correspond to a state where infections are caused by variants with relatively similar genetic attributes. When applied to the duration period of a wave, these respective metrics are referred to as *wave-entropy* and *wave-heterogeneity* in the rest of this section.

**Fig. 2.**
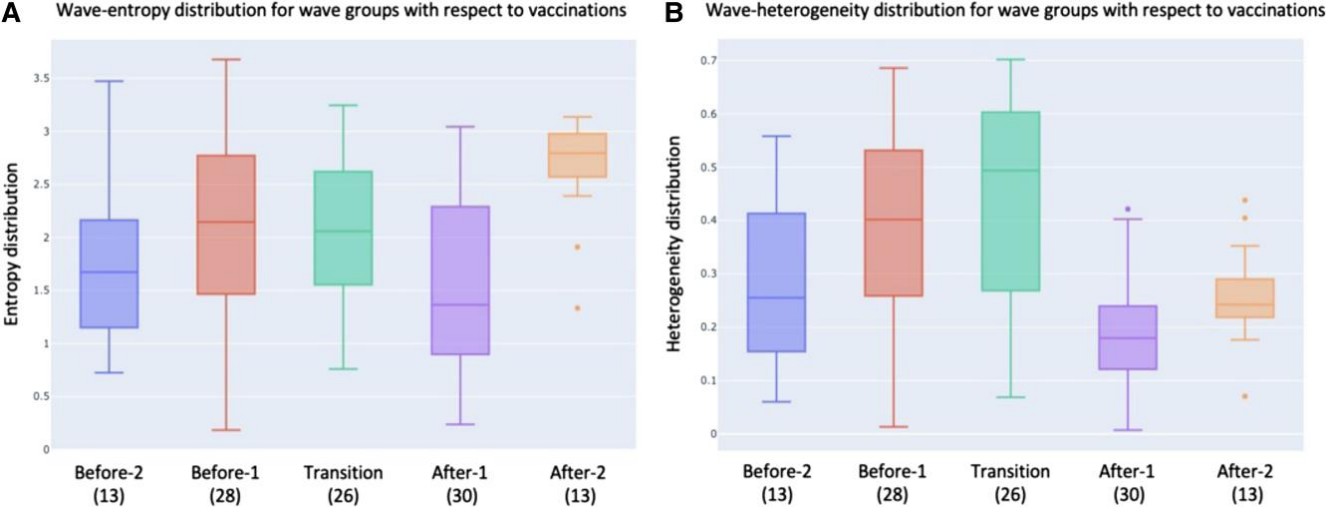
Wave-entropy and wave-heterogeneity distributions. Distribution of wave-entropy A) and wave-heterogeneity B) values in five distinct sets of waves: Before-2 (13 waves) and Before-1 (28 waves) including, respectively, last and second last waves ending prior to start of the country vaccination campaign; Transition (26 waves) including waves that started before the country vaccination campaign but ended afterwards; After-1 (30 waves) and After-2 (13 waves), including the first and second waves, respectively, that started after the start of the country vaccination campaign.

Given the potential impact of mass-vaccination campaigns against SARS-CoV-2 on the evolution of variants’ diversity, the study analyzes the temporal trends in wave-heterogeneity and wave-entropy before and after country vaccination campaigns started. Figure 2A and B displays the distribution of wave-entropy and wave-heterogeneity values, respectively, within five distinct groups of waves. The first two groups, *Before-1* and *Before-2*, include the second-to-last and last waves, respectively, ending prior to the start of the country vaccination campaign. The third group, *Transition*, includes waves that started before the country vaccination campaign but ended after it started. Finally, the last two groups, *After-1* and *After-2*, include the first and second waves that started after the start of the country vaccination campaign. Overall, across all 30 countries, there are 13 waves in the Before-2 group, 28 waves in the Before-1 group, 26 waves in the Transition group, 30 waves in the After-1 group, and 13 waves in the After-2 group. The statistical significance of potential differences in distributions between each consecutive group of waves has been assessed via a *t*-test with the 0.05 threshold (43). This threshold has then been adjusted with the Holn–Bonferroni method to counteract potential issues of testing multiple hypotheses simultaneously (44) (see Materials and methods for more details).

The results highlight that wave-entropy distributions remain statistically similar in the Before-2, Before-1, and Transition waves with median values of 1.67 (q1: 1.15, q3: 2.16), 2.14 (q1: 1.47, q3: 2.77), and 2.06 (q1: 1.55, q3: 2.62), respectively. However, there is a significant decrease in wave-entropy from Transition to After-1 waves having a median value of 1.36 (q1: 0.90, q3: 2.29) (*P* = 0.02), before a statistically significant increase from After-1 to After-2 waves with a median of 2.79 (q1: 2.57, q3: 2.98) (*P* = 6.5 × 10^−5^). The 105% increase in wave-entropy from After-1 to After-2 waves is significant even when accounting for multiple hypotheses testing as the lowest Holn–Bonferroni adjusted *t*-test threshold is 1.3 × 10^−2^ for four tests. Note also that the variance of wave-entropy of the After-2 waves is significantly lower as the difference between the first and third quantiles is significantly smaller.

Wave-heterogeneity values increase, with low statistical significance, from the Before-2 to Before-1 waves, from a median of 0.26 (q1: 0.15, q2: 0.41) to 0.40 (q1: 0.26, q2: 0.53) (*P* = 0.07), then remain at statistically similar levels from Before-1 to Transition waves with a median for the latter of 0.49 (q1: 0.27, q2: 0.60). However, there is a statistically significant decrease in variants’ heterogeneity from Transition to After-1 waves, with a median of 0.18 (q1: 0.12, q3: 0.24) (*P* = 7.6 × 10^−7^), and then an increase with low statistical significance from After-1 to After-2 waves, with a median of 0.24 (q1: 0.21, q3: 0.29) (*P* = 0.10). The 63% decrease in wave-heterogeneity from Transition to After-1 waves also remains significant when accounting for multiple hypotheses testing.

The evolution of wave-entropy and wave-heterogeneity from Before-2 to Before-1 waves could potentially be explained by the immunity effects of previous natural infections in the population on the ability of variants to spread. In the Transition group, vaccination campaigns typically started toward the end of the wave, with a median of 109 days (q1: 28, q2: 154) after the wave started. Consequently, the evolution from Before-1 to Transition waves is not likely to be significantly impacted by vaccinations when compared with the natural immunity acquired by infections. However, the evolution from Transition waves to the After-1 waves is potentially impacted by both natural and vaccine-induced immunity. Similarly, natural and vaccine-induced population immunity may affect the evolution of the wave heterogeneity and entropy metrics from the After-1 to the After-2 waves, but with a longer time elapsed since vaccinations and thus showing a more prominent effect.

The decrease in wave-heterogeneity and wave-entropy from Transition to After-1 waves is potentially supporting the hypothesis that vaccination creates an additional immune pressure that reduces variants diversity, perhaps because a large fraction of the population is exposed over a short period of time to the same immune stimuli. In particular, such an evolution in variants’ genetic diversity could be explained by the nature of the current SARS-CoV-2 vaccines that generate neutralizing antibodies based on a reference strain of the virus. Subsequently, it can be expected to see a more homogeneous immune pressure that favors those variants with enough vaccine-escape mutations (45, 46). Yet, wave-heterogeneity and wave-entropy ultimately increase in the After-2 waves which could potentially be explained by the emergence of vaccine-escape variants, as well as the waning vaccines’ protection against infections over time, resulting from the waning of the vaccine-induced antibodies (47), combined with the spread and diversification of the vaccine-escape variants. The continuous emergence of new variants and their genetic diversification underscores the need for additional methods to monitoring variants and early detection of the ones that are more likely to spread.

### Machine-learning-enabled early-stage prediction of variant spread trajectory

The descriptive analyses presented above can be leveraged to develop a machine-learning-enabled risk assessment model to predict the spread trajectory of each variant in each country, as early as 1 or 2 weeks, after their first detection.

There are multiple existing approaches aiming to develop risk assessment methods to better understand and promptly respond to emerging infectious viruses such as SARS-CoV-2. Most of these methods rely on analyses of the mutations of each SARS-CoV-2 variant, and their genetic functionality. For example, reverse genetics methods are used to analyze the phenotypic consequences of new variants’ mutations and their impact on the affinity of the viral spike protein for cellular receptors (48). Other approaches study genes expression in immune cells to determine the level of susceptibility and severity of a new variant (49). However, one limitation of reverse genetics and gene expression method protocols is that they typically take multiple days, up to 5 days, to perform, and require biosafety level 3 facility which could hamper their speed and availability (50, 51). New methods have been designed to overcome these limitations, such as the use of virus-like particles in the assessment of SARS-CoV-2 mutations necessitating a lower level of biosafety (52). Still, even for these more recent methods, the rapid and accurate assessment of the virulence and transmissibility of new variants remains a critical scientific challenge (50).

Parallel to these approach, traditional epidemiological models, such as the susceptible-infected recovered model and its different extensions, have been further enriched with multiple additional inputs beyond the traditional input of infection counts time series (18). These additional inputs include among others, mobility or contact tracing data, and require more advanced computational power to calibrate the respective models (53). Yet, these methods still face technical challenge such as parameter estimation and have limited long-term predictive power. Moreover, they do not differentiate between variants which could result in further variability in the accuracy of their predictions (54, 55). More recent methods used by the CDC aim to forecast the variants’ relative proportions within a 2- to 3-week prediction window (9). However, reliable predictions of the longer term spread trajectory of new variants, and whether they will cause a significant number of infections, still need to be developed. The newly proposed model tackles this challenge and incorporates features related to infection trajectory, nonpharmaceutical interventions, as well as genetic features. The latter are motivated by the descriptive analysis presented about the genetic properties of dominant variants, as well as more generally, the variants’ genetic characteristics within and across waves. More specifically, it adapts the measures outlined in the descriptive analysis, and assesses their predictive power to identify early on, which variants will spread and cause a high number of infections.

The dataset used to develop the machine-learning model includes variants from 30 countries from 2020 February 1 to 2022 March 19. Each observation consists of a pair of a variant and a country, with a total of 3,506 observations. The features, or independent variables, of each variant–country observation are assessed after an *observation period* defined as 1 or 2 weeks after the first detection of the new variant in the respective country, and the categorical dependent variables correspond to whether the variant was *infectious* during the 3 months since its detection in the country (see detailed definitions and discussion below). The 3-month prediction horizon is motivated by the fact that for variants that evolve to be dominant in a future wave, it typically takes 1 to 3 months from their detection until the peak of the wave, with a median of 63 (q1: 28, q3: 112) days. However, the vast majority of variants cause very low levels of infections, and in fact, only a few causes a very high relative number of infections, which makes it challenging to develop early prediction spread trajectory and related risk assessment capabilities.

The definition of an infectious variant is motivated by the density distribution of cases caused by variants in every country 3 months after their detection. Specifically, only 8.3% of them caused >1,000 cases per million after 3 months. Thus, the developed machine-learning model attempts to detect the infectious variants that are defined as ones that caused >1,000 cases per million people in the following 3 months after their first detection. Specifically, the dependent variable values of each observation of a variant–country pair is 1, if the variant turned out to be infectious and 0 otherwise (see Fig. S2 for a sensitivity analysis on the threshold of infectious variants and Fig. S3 for the full density distribution of cases per variant–country).

**Fig. 3.**
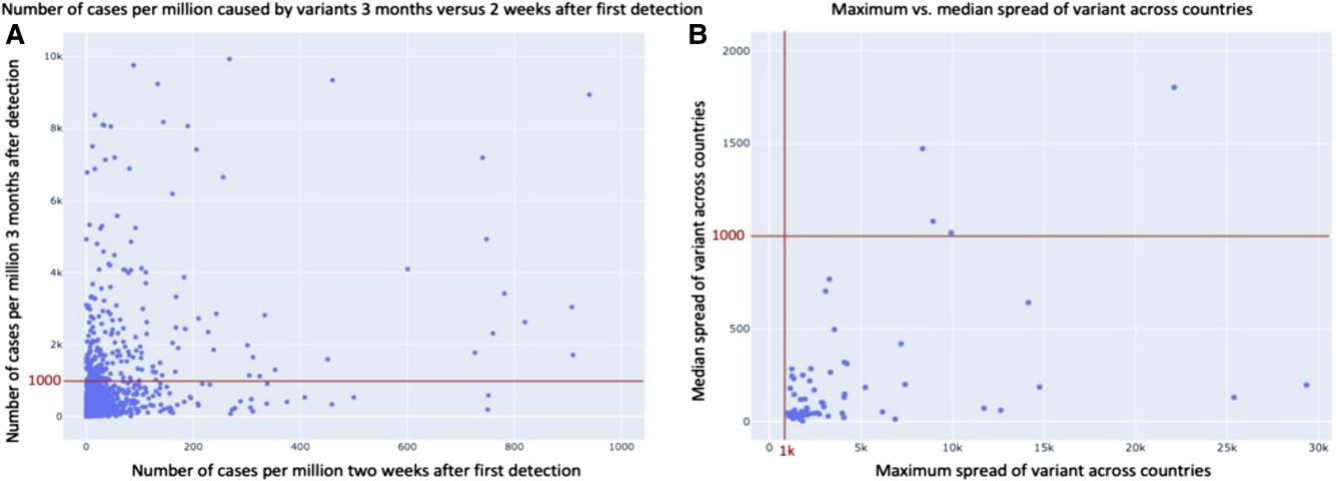
Distribution of infection cases per variant and country. A) Comparison of the number of cases 2 weeks after first detection to 3 months afterwards for all the variants and countries under study. B) Comparison of the maximum and median spread across countries after 3 months of variants that were infectious in at least one country.

Figure 3A plots the joint distribution of the number of cases per million caused by variants, 2 weeks, and 3 months after their detection. It highlights that the majority of variants, including the most infectious ones, are still causing a relatively small number of infections within 2 weeks after their detection, with an overall median of 2.5 (q1: 0.56, q3: 10.6) cases per million. In addition, variants having similar levels of infections after 2 weeks following their detection can have a very different trajectory after 3 months, either decaying or emerging to cause many infections. Figure 3B plots the joint distribution, across all variants that were infectious in at least one country, of the maximum and median spread of a variant across countries after 3 months. It shows that among 96 variants that were detected in multiple countries and have been defined as infectious in at least one of them, only 4 have a median spread above the definition threshold of 1,000 infections per million people. That is, most of them (92 out of 96) do not qualify the definition of being infectious in at least half of the countries, where they have been detected. Hence, a variant can be defined as infectious in one country, and at the same time could have a very different spread trajectory in other countries (see Fig. S5 for these figures in logarithmic scale and Table S3 for summary statistics on the spread of variants across countries). This second observation is in line with phylogenetic evidence showing a geographic specification of the virus mutational evolution over time (12). Thus, early-stage prediction of infection trajectory in a country is nontrivial, even if the variant has already appeared in other countries. This highlights the importance of models that incorporate additional and country-level hypothesized predictive features.

#### Genetic features

Mutations in the different virus proteins have a critical impact on its transmissibility (56–58), vaccine-escape potential, and reinfection risk (59, 60). Thus, the study uses the metrics introduced in the descriptive sections above to design genetic features that could be predictive of their infectivity level. First, the study uses the Jaccard distance to define the *week-distance* feature that measures how different the mutations of a new variant are from those of the first dominant variant during the 1-week (resp. 2 weeks) observation period. This feature would potentially point out immune-escape variants that have a very different profile of mutations from most circulating variants. Moreover, the model applies variants’ entropy and heterogeneity metrics to the observation period, 1 or 2 weeks, of each new variant. These features are called, respectively, *week-entropy* and *week-heterogeneity*. They characterize the genetic diversity among variants circulating during the observation period as it could potentially enable the emergence of a new variant with mutations that could favor its immune-escape potential or transmissibility (39). Moreover, the number of mutations in each of the virus proteins have also been calculated and included as potential predictive features.

#### Infection trajectory features

Traditional epidemiological models typically predict the trajectory of SARS-CoV-2 infections based on the patterns of its temporal evolution, considering properties of the respective time series of the number of infections caused by the variant over time (19, 61, 62). Moreover, variants compete against each other on their respective share of infections (63, 64). Hence, the proposed model includes features about the weekly evolution of the proportion of reported sequences for the variant among all variants actively circulating in the respective country at the time of detection of the new variant. Additionally, the model estimates the weekly number of new infections caused by the variant as the product of the total number of new infections by the proportion of the respective variant among all others. The model feature set also includes variables that capture the maximal number of cases per million caused by the variant in other countries up to the end of the observation period.

#### Pharmaceutical and nonpharmaceutical intervention features

Additional features included in the model measure the stringency of social distancing restrictions and vaccinations to account for their potential impact.

The model feature set consists of 31 potentially predictive variables that comprehensively capture the genetic attributes of a new variant, its early spread trajectory, as well as the nonpharmaceutical public health and vaccination interventions during the time in which the respective variant emerged (see Materials and methods and Table S4 for a detailed definition of all the features used). These features are used to predict how infectious the variant would be, using a supervised machine-learning framework. Specifically, the newly developed models leverage interpretable machine-learning methods with a tree-based gradient boosting algorithm (65). Overall, two models are developed to predict whether a new variant will be infectious with >1,000 cases per million during the 3 months following the observation period which is either 1 week or 2 weeks after its first detection in the respective country. The models were trained on the variants detected up to 2021 April 1, and their performance was evaluated through out-of-sample tests on variants detected after 2021 April 1, and throughout 2022 January 1.

Table 1 summarizes the predictive performance in detecting the variant–country instances that will have >1,000 new cases per million in the next 3 months. The models achieve an out-of-sample AUC 86.3% after 1-week observation period, and the performance improves with a longer 2-week observation period to 90.8%. Given the imbalance in the prediction, it is also important to assess the sensitivity and the specificity of the model to the relatively infrequent detection of infectious variants in some countries. The models associate a risk score to each variant in each country, and the threshold used to turn this score into a binary prediction is set up in the training set to maximize a weighted sum of sensitivity and specificity, with sensitivity being weighted as three times the specificity to favor the detection of infectious variants even at the cost of having false positives. In particular, it can detect 72.8% of the variants that will cause a wave of at least 1,000 cases in the next 3 months after an observation period of only 1 week. This performance increases to 80.1% with 2-week observation period. At the same time, the models remain specific and identify most of the variants causing a relatively small number of cases up to 82.5% after 1 week and 85.9% after 2 weeks.

**Table 1.** Results of the out-of-sample performance of original models.

Observation period	AUC (%)	Sensitivity (%)	Specificity (%)	Accuracy (%)
1 week	86.3	72.8	82.5	81.6
2 weeks	90.8	80.1	85.9	85.4

Out-of-sample performance of original models for predicting variants which will cause >1,000 new cases per million in 3 months after their detection in every country.

Additionally, for every observation period, the model performance has been compared with a reference model that only considers infection trajectory features and excludes all the genetic and relative proportion features of the variant. As can be seen in Table 2, the sensitivity of the model drops significantly to 59.5% (resp. 67.3%) for the 1-week (resp. 2-week) observation period. More careful analysis suggests that these reference models classify most variants as noninfectious to maintain a high level of accuracy at the cost of missing infectious ones. This underscores the importance of the new genetic features introduced in addition to trajectory features commonly used, as well as the cross-country features.

**Table 2.** Results of the out-of-sample performance of models without genetic and relative proportion features.

Observation period	AUC (%)	Sensitivity (%)	Specificity (%)	Accuracy (%)
1 week	84.0	59.5	90.3	87.5
2 weeks	90.4	67.3	92.6	90.4

Out-of-sample performance of models without genetic and relative proportion features for predicting variants which will cause >1,000 new cases per million in 3 months after their detection in every country.

**Fig. 4.**
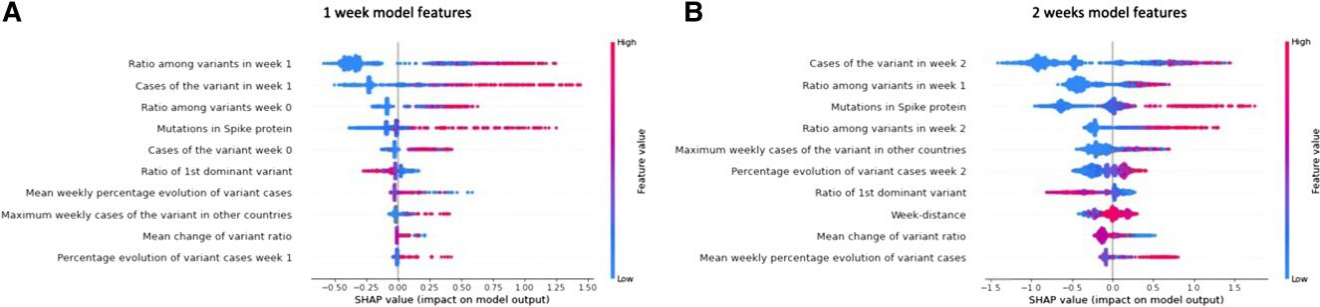
Shapley values of top 10 most predictive features. Shapley values of the top 10 features identifying infectious variants for the 1-week A) and 2-week B) models that predict variants likely to cause >1,000 cases per million in the next 3 months. The features are ranked based on their predictive power from the top to bottom.

In order to identify the risk drivers and main predictive features of infectious variants, the study performs a Shapley analysis which is a common approach to quantify the marginal predictive power of each feature (66). Figure 4 displays the results of the Shapley impacts of the top 10 most predictive features for the 1week and 2-week observation models that predict variants likely to cause >1,000 cases per million in the next 3 months. The other features are not displayed but still may contribute to the overall predictive capabilities of the models. In the names of the features displayed in the figure, week 0 corresponds to the week of detection of the new variant. Then, the variant’s early trajectory is observed during the first week, referred to by week 1, and potentially the second week, referred to by week 2, following its detection. This analysis shows that the ratio of the sequences of the new variant among all other circulating variants, and the number of infections caused by this variant at the time it has been detected and the observation period are the strongest risk predictors. This outcome is partially similar to traditional epidemiological models that typically calibrate their respective parameters based on the past trajectory of number of infections count (19, 61), but adds the dimension of how dominant the variant is. Considering the genetic features, the models identify mutations in the spike as the third most important risk predictor, which is in line with previous research linking spike mutations to the virus infectivity (48, 67, 68). This result also aligns with parallel research efforts that leverage machine learning to predict alarming future spike mutations, which also observe significant mutational changes in the spike protein between successive variants of concern (69). Similarly, the higher the week-distance feature is, the more likely it is for the variant to widely spread. This could be explained by hypothesis that in order to evade the natural immunity induced by the prior dominant variant, the new variant has to acquire a sufficiently large number of new genetic mutations. This will allow the variant to target new subgroups of the population that were not exposed or had natural immunity to the prior dominant variant (70–72). In contrast, neither restrictions on gatherings and public events nor vaccination rates have been empirically identified among the top 10 risk drivers of becoming a dominant variant.

## Discussion

Using extensive analysis of the genetic sequences of SARS-CoV-2 variants and their historical trajectories, the paper highlights several important characteristics that drive the infectivity of new variants which ultimately can lead to new infection waves. This analysis relies heavily on a notion of “genetic distance” using the Jaccard distance metric to compare mutational variations between successive infection waves, as well as their diversity within an infection wave. Descriptive analysis of the infection waves over time shows that every wave of infection is driven by a small number of new variants that cause almost all infections during the wave, and are typically genetically very distant from previously dominant variants. Analysis of the within wave variant diversity reveals that it decreased immediately after the start of the vaccination campaigns, and then increased again. This provides potential hints as to the impact of natural and vaccine-induced immunity on variants’ diversity.

The newly developed predictive models are able to identify infectious variants as early as 1 week and 2 weeks after their first detection with an out-of-sample AUC of 86.3 and 90.8%, respectively. The strongest predictive signal features include the early trajectory of the infections caused by the variant, its spike mutations, and the week-distance from the dominant variant during the observation period. These findings are consistent with prior epidemiology research that uses historical infection trajectory to assess its future evolution, as well as with prior genetic analysis of SARS-CoV-2 variants suggesting that the infectivity of variants is driven by the spike protein. Moreover, these results support the hypothesis that the infectious new variants are those that acquire enough mutations which either can lead to reinfections or enable targeting new subgroups of the population that were naturally immune to previous variants. The superior predictive performance relative to traditional models, that are based only on infection trajectory features, highlights the importance of including genetic features to obtain more sensitive models. Notably, as a future research direction, it would be interesting to further explore how genetic and biological understanding of the infectivity and spread of variants can be translated into hypothesized predictive features that would be assessed on data. Additionally, the ultimate performance of predictive models is evaluated in the context of the decisions it is used to inform. Thus, future work will be able to fine-tune the models based on the decision maker’s preferences, such as the tradeoff between sensitivity and precision.

This study has some limitations. It assumes that all variants are sampled and reported proportionally to the number of cases they cause. This may not be the case due to variable surveillance capacity over time and across countries. However, as surveillance capacities are increasing, this limitation is likely to play a substantially smaller role in the future. Moreover, the distance metric, used to calculate how different variants are from each other, only considers the number of distinct and overlapping mutations, each variant carries, but does not account for the potential functional impact of each mutation, nor for the mutation rate among different variants.

In summary, this work provides an analytical framework that leverages multiple data sources, including genetic sequence data and epidemiological data via machine-learning models to provide improved early signals on the spread risk of new SARS-CoV-2 variants. It calls for more research that makes use of genetic sequencing to enhance spread predictions and the risk assessment of new variants. Moreover, this approach could potentially be extended to other respiratory viruses such as Influenza, Avian Flu viruses, or other Corona viruses.

## Materials and methods

### Experimental design

The study starts with a descriptive analysis of infection waves and variants’ evolution. It provides a formal definition of an infection wave and analyzes the distribution of variants in every wave. From there, it defines the first, second, and third dominant variants as those with respectively the first, second, and third highest ratios of reported sequences during each wave. Then, the variants are characterized through several genetic features based on their mutations across the different proteins, and specifically, how different they are from one another. This is captured through the Jaccard distance induced on the set of mutations each variant has with respect to the Wuhan variant. Leveraging the metric between variants, the paper introduces several metrics to describe waves of infections. The interwave distance metric measures how different the dominant variant in the “current” wave is from variants circulating in the previous wave, and the wave-entropy and wave-heterogeneity metrics measure the diversity of variants circulating during each wave. The following section presents the precise mathematical definition of all the features used in the descriptive analysis.

The second part of the study develops a predictive model that for each new variant detected in a given country, predicts based on an observation period of 1 to 2 weeks after the variant detection, whether the variant is likely to be infectious, i.e. to cause 1,000 cases per million in the next 3 months. For this purpose, the study adapts some of the metrics designed in the descriptive part and complements them with additional features that could potentially be predictive. This work creates then a rich set of independent variables that are used to calibrate and train a machine-learning algorithm to perform the prediction.

### Design of descriptive features

#### Number of mutations

These features capture the genetic mutations of the current variant compared with the base Wuhan strain (GISAID reference: EPI-ISL-402124). Let *M*(*i*) and *M*_P_(*i*) be, respectively, the set of all genetic mutations of variant *i* with respect to the Wuhan strain, and those that specifically occur within the protein P. The cardinality of these sets is defined as *N_i_* = |*M*(*i*)| and *N_i, p_* = |*M*_P_(*i*)|.

#### Jaccard distance

This distance is computed as the percentage ratio between the total number of mutations in all the proteins that are unique to exactly one of the variants and the total number of mutations across the pair. The Jaccard distance receives values from 0, when all mutations are identical, to 1 for completely disjoint sets of mutations. The higher this distance is between two variants, the more different they are. For variants *i* and *j*, the Jaccard distance is


$$J(i,j)=1-\frac{\left|M(i)\cap M(j)\right|}{\left|M(i)\cup M(j)\right|}$$

#### Interwave distance

This metric corresponds to the weighted average distance of the dominant variant of a given “current” wave from the variants reported during the previous wave with weights being the reported sequences ratios of every variant in the previous wave. Let *W*_1_ be the set of variants in wave 1, *r_i_* be the ratio of reported sequences for every variant *i* in *W*_1_. Similarly, let *W*_2_ be the set of variants in the subsequent wave, and denote by *j* the first dominant variant in *W*_2_. The interwave distance between *W*_1_ and *W*_2_ is defined as


$$D(W_{1},W_{2})=\underset{i\;\in \;W_{1}}{\sum }\;r_{i}\;J(i,j)$$

#### Wave-heterogeneity

This metric corresponds to the weighted average of Jaccard distances across all pairs of variants circulating during a wave denoted by *W*, with weights being the product of the ratios of reported sequences for the respective pair of variants in the wave. Specifically, for every pair of variants (*i*, *j*) in a wave *W*, their Jaccard distance is weighed by the product of the respective proportions of genetic sequences, denoted by *r_i_* and *r_j_*. Then, the wave-heterogeneity metric of *W* is defined as


$$H(W)=\frac{1}{2}\underset{i,j\;\in \;W}{\sum }\;r_{i}\;r_{j}\;J(i,j)$$

#### Wave-entropy

This metric is inspired by the concept of entropy in statistical physics (40) and measures the degree of dispersion of the variants circulating during the observation period. The wave-entropy is computed as the negative of the product between the variant’s proportion among reported sequences for all variants during the wave and the logarithm of this ratio, summed over all variants. Specifically, let *W* be the set of variants during the wave, and *r_i_* be the ratio of reported sequences of every variant *i* in *W*. Then, the wave-entropy is defined as


$$E(W)=-\underset{i\in \;W}{\sum }\;r_{i}\;\log(r_{i})$$

### Design of predictive features

The study adapts and extends the new features described in the descriptive analysis, to obtain the following potentially predictive features.

#### Week-distance

This feature measures the Jaccard distance of the variant under study from the first dominant variant during the 1-week (resp. 2-week) observation period.

#### Week-heterogeneity

This feature is analogous to the wave-heterogeneity metric, and is calculated, considering the variants circulating during the observation period, 1 or 2 weeks, and their relative proportion of reported sequences during this period.

#### Week-entropy

This feature is analogous to the wave-entropy metric, and is calculated, considering the variants circulating during the observation period, 1 or 2 weeks, and their relative proportion of reported sequences during this period.

#### Early infection trajectory features

These features capture the dynamic evolution of the variant in terms of infection cases and distribution among other variants. They have been computed for every new variant in every country. In the definition of the following features, week 0 corresponds to the week of detection, then week 1 and week 2 correspond to the first and potentially the second week of the observation period.

- The weekly proportion of the variant’s number of reported sequences among reported sequences for all variants circulating in the respective country during the week when the new variant has been detected, week 0, then in each week of the following observation period, week 1 and week 2.
- Weekly number of infection cases for the variant *i* in the week of its detection, week 0, then in each week of the observation period, evaluated as the ratio of the sequences reported for this variant times the total number of cases reported.
- Maximum weekly number of infection cases caused by this variant in the other countries up to the end of the observation period.
- Percentage evolution of the proportion of the variant’s reported sequences among all other variants in the country from week 0 to week 1, then from week 1 to week 2.
- Mean percentage evolution of the ratio of the variant’s reported number of sequences among all other variants in the country for the 2 weeks’ model.
- Mean second derivative of the ratio of the variant’s reported number of sequences to characterize the mean acceleration of its spread relatively to other variants.
- Percentage evolution of the variant’s weekly number of infections from week 0 to week 1, then from week 1 to week 2.
- Mean weekly percentage evolution of the variant’s number of infections for the 2 weeks’ model.
- Mean second derivative of the variant’s weekly number of infection cases to characterize the mean acceleration of its spread in terms of infections.

### Pharmaceutical and nonpharmaceutical interventions

- The study includes features that indicate restrictions on social gatherings, that evaluate from 0 to 4 the limit on gathering sizes (0—no restrictions: 1—restrictions on very large gatherings [the limit is above 1,000 people]; 2—gatherings between 100 and 1,000 people; 3—gatherings between 10 and 100 people; 4—gatherings of <10 people); and cancellation measures on large public events (0—no measures, 1—recommended cancellations, 2—required cancellations) (73).
- The feature set also includes the vaccination rate for every new variant in every new country, corresponding to the proportion of the population having at least one dose from any kind of SARS-CoV-2 vaccine (5).

### Multiple hypotheses testing for wave-heterogeneity comparison

In the final part of the descriptive analysis, infection waves have been partitioned into several time-ordered groups, including Before-2, Before-1, Transition, After-1, and After-2. The study then compared the distributions of values of waves-entropy and wave-heterogeneity metrics across successive pairs of groups with the 0.05 threshold to validate the hypothesis of a significant difference between the groups (43). To control for multiple hypotheses testing, the Holn–Bonferroni method (44) was used. Given a threshold value *α*, here 0.05, the *P*-values of the *N* tests, here *N* equals 4, are ranked from lowest to highest, then the adjusted threshold for the test with ranked *P*-value *k* is $\frac{\alpha }{N+1-k}$. In this work, the lowest adjusted threshold has a value of 1.3 × 10^−2^ and validates the significance of the decrease in wave-heterogeneity from Transition to After-1 waves, and the increase in wave-entropy from After-1 to After-2 waves.

### Machine-learning model

All of these features have been calculated for every variant in every country and used to calibrate and train a machine-learning algorithm. The study uses a binary classification gradient boosting model (65, 66) to predict the probability of the variant in a specific country to cause >1,000 cases per million. The training set corresponds to the variants observed in each country up to 2021 April 1 and the test set corresponds to those observed from 2021 April 1 to 2022 January 1, as it needs to evaluate the cases they caused 3 months afterward. The model’s hyperparameters and out-of-sample performance were tuned and calculated, respectively, through 3-fold cross-validation on the training set. Specifically, it first divides the whole training set into three stratified folds, creating three different splits where 1-fold is selected as the test set and the four others as the training set. After the hyperparameter tuning, the optimal threshold value, which categorizes predicted probabilities as binary outcomes, is selected as the value that maximizes the sum of the model’s in-sample sensitivity and specificity. The weight of sensitivity has been set as 3 times the weight of specificity to favor the detection of infectious variants even if this implies making more errors on the noninfectious ones.

## Supplementary Material

pgad424_Supplementary_Data

## Data Availability

This work leverages data from GISAID (1), which is the largest open database of collected genetic sequences of SARS-CoV-2 with 9.5 million sequences collected across the world as of 2021 March 19. Each observation corresponds to a genetic sequence of the virus reported by a specific country at a specific date. It uses the Wuhan hCoV-19/Wuhan/WIV04/2019 as a reference and from there describes all the genetic mutations of each sequence, including its Pango lineage (20). The work also uses data about reported SARS-CoV-2 cases, vaccinations, and tests reported by each government and gathered in Hannah et al. (5). The study uses data about cancellation of public events and restrictions on public gatherings (73). The computer code and materials are available at: https://github.com/ElghaliZ/COVID19Variants
