# Supplementary material for: Predicting the spread of SARS-CoV-2 variants: An artificial intelligence enabled early detection
Source: PNAS Nexus. 2024 Jan 2;3(1):pgad424. doi: 10.1093/pnasnexus/pgad424 (PMC10759796; doi:10.1093/pnasnexus/pgad424)
Supplement: pgad424_Supplementary_Data [file pgad424_supplementary_data.docx]

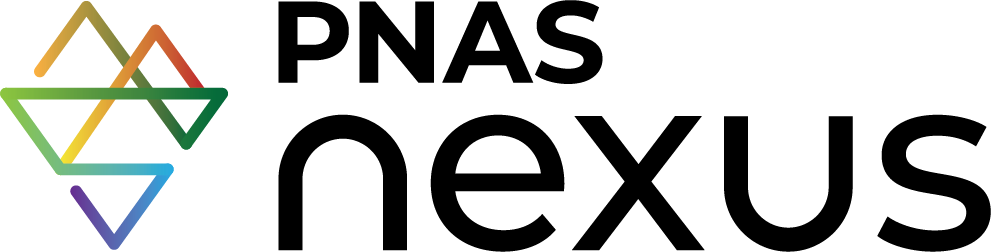


**Supporting Information for:**

**Predicting the Spread of SARS-CoV-2 Variants: An AI-Enabled Early Detection**

# Retsef Levi^1^*, El Ghali Zerhouni^2^, Shoshy Altuvia^3^

^1^Sloan School of Management, Massachusetts Institute of Technology, Cambridge, USA

^2^Operations Research Center, Massachusetts Institute of Technology, Cambridge, USA

^3^Department of Microbiology and Molecular Genetics, The Hebrew University-Hadassah Medical School, Jerusalem, Israel

*Corresponding author: Prof. Retsef Levi, Sloan School of Management, Massachusetts Institute of Technology, 100 Main Street, E62-562, MA 02142, USA, **Tel:** 617-253-4155, **Email:** [retsef@mit.edu](mailto:retsef@mit.edu)

**This PDF file includes:**

Figures S1 to S5

Tables S1 to S10


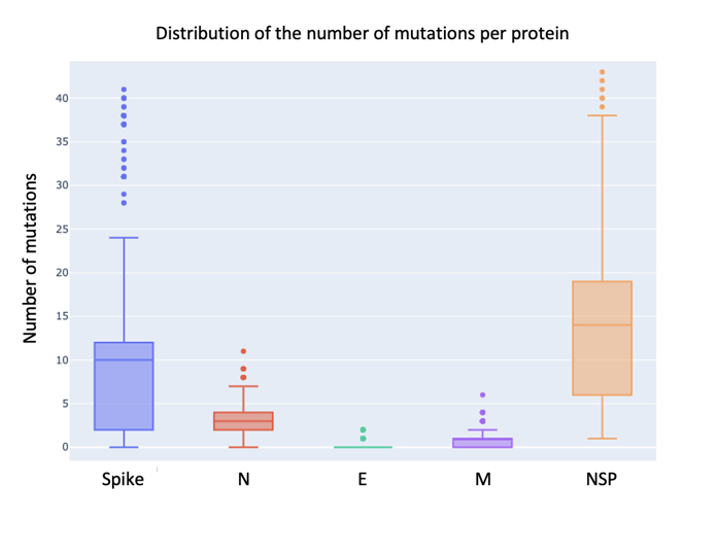


Fig. S1. Distribution of the number of mutations per protein. Number of mutations in the reported variants in every country relative to the Wuhan strain for the spike, N, E, M, and NSP Proteins. In addition to the Spike, N, and NSP mutations, protein M has a median of 1 mutation (q1: 0, q2: 1), and protein E has very few mutations, with no mutations in 92.7% of the variants, 1 mutation in 7.0% of them and 2 mutations in the rest.


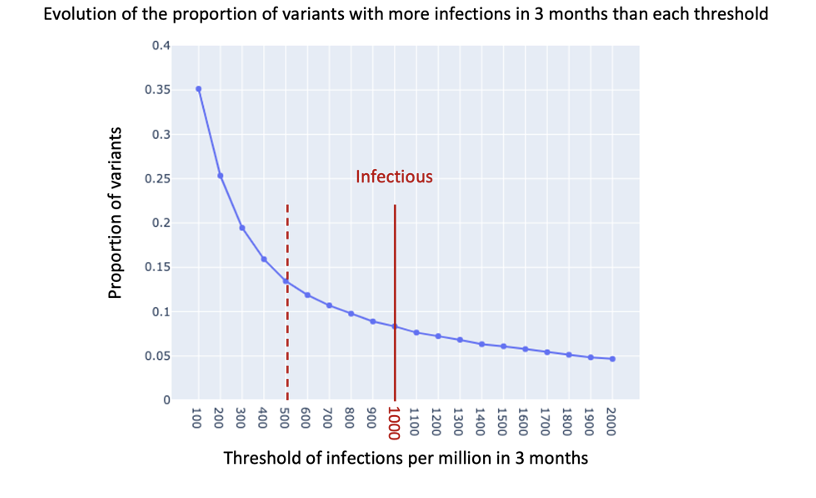


**Fig. S2. Distribution of the number of cases per variant**. It shows a sensitivity analysis of the proportion of variants causing more infections in 3 months than each threshold considered from 100 to 2000 infections per million. In this work, the threshold of 1000 cases per million has been selected as the threshold for infectious variants, as it corresponds to the inflection point in the curve. In order to test the robustness of the results, the study also uses half this value, 500 cases per million, as the threshold for infectious variants in the predictive models.


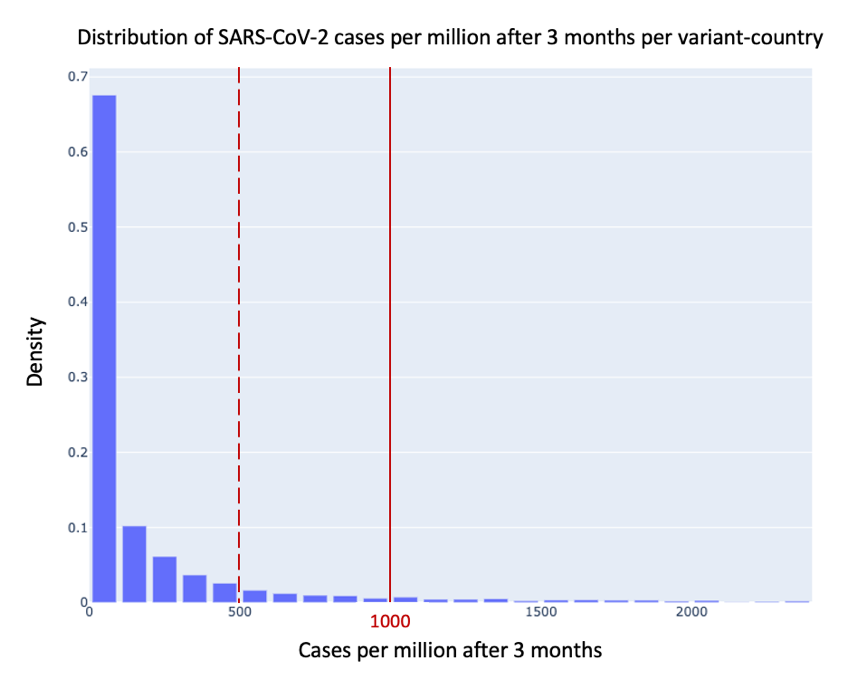


**Fig. S3. Distribution of the number of cases per variant-country**. It presents a density distribution of the number of cases caused by variants in each country 3 months after their respective detection. It shows that the majority of variants are not infectious in the countries where they are detected, as only 8.3% (resp. 13.4%) spread caused more than 1000 cases per million (resp. 500 cases per million).

Table S1. Data profile of SARS-CoV-2 variants per country. Table S1 presents the data profile for the 30 countries considered in the study. It includes the number of genetic sequences collected, the number of variants detected, and the number of variants that caused more than 500 cases, then 1000 cases, per million in their respective countries 3 months after their first detection.

| Country | Number of genetic sequences | All variants | More than 500 cases/million in 3 Months | More than 1000 cases/million in 3 Months |
| --- | --- | --- | --- | --- |
| USA | 2947246 | 519 | 15 | 9 |
| United Kingdom | 2460836 | 436 | 22 | 11 |
| Germany | 462409 | 213 | 14 | 10 |
| Denmark | 421395 | 208 | 25 | 17 |
| Canada | 298939 | 187 | 14 | 9 |
| France | 266116 | 158 | 25 | 12 |
| Japan | 236105 | 61 | 2 | 2 |
| Sweden | 173386 | 122 | 17 | 13 |
| India | 167536 | 119 | 4 | 0 |
| Switzerland | 131941 | 150 | 33 | 20 |
| Brazil | 124776 | 57 | 11 | 9 |
| Spain | 114303 | 104 | 23 | 12 |
| Italy | 111413 | 110 | 18 | 14 |
| Netherlands | 108516 | 106 | 29 | 17 |
| Austria | 101696 | 49 | 9 | 6 |
| Belgium | 96081 | 115 | 27 | 17 |
| Turkey | 88168 | 75 | 14 | 9 |
| Australia | 80406 | 58 | 6 | 4 |
| Poland | 78275 | 67 | 15 | 8 |
| Ireland | 59034 | 69 | 24 | 15 |
| Slovenia | 57901 | 54 | 24 | 20 |
| Mexico | 56549 | 55 | 8 | 5 |
| Israel | 53857 | 65 | 20 | 15 |
| Norway | 53727 | 77 | 18 | 9 |
| South Korea | 39680 | 23 | 5 | 4 |
| Lithuania | 35675 | 36 | 15 | 15 |
| South Africa | 34290 | 39 | 13 | 7 |
| Portugal | 29766 | 64 | 25 | 17 |
| Finland | 28932 | 48 | 13 | 10 |
| Luxembourg | 25456 | 61 | 24 | 13 |

Table S2. Waves with a successively dominant variant. Detailed information about the waves that have the same 1^st^ dominant variant compared to the previous wave in their respective country.

| Country | Wave start date | Wave end date | Dominant variant | Ratio in current wave | Ratio in previous wave |
| --- | --- | --- | --- | --- | --- |
| USA | 2020-05-31 | 2020-09-13 | B.1 | 0.24 | 0.54 |
| Germany | 2021-02-21 | 2021-06-13 | B.1.1.7 | 0.84 | 0.24 |
| Denmark | 2021-04-11 | 2021-06-27 | B.1.1.7 | 0.96 | 0.32 |
| Sweden | 2021-02-07 | 2021-06-20 | B.1.1.7 | 0.82 | 0.18 |
| Switzerland | 2021-02-28 | 2021-06-06 | B.1.1.7 | 0.89 | 0.16 |
| Netherlands | 2021-02-14 | 2021-06-20 | B.1.1.7 | 0.86 | 0.22 |
| Turkey | 2020-07-19 | 2020-10-04 | B.1.1 | 0.43 | 0.42 |
| Belgium | 2021-02-21 | 2021-06-13 | B.1.1.7 | 0.76 | 0.31 |
| Slovenia | 2020-12-20 | 2021-03-07 | B.1.258.17 | 0.76 | 0.40 |
| Portugal | 2020-05-24 | 2020-08-09 | B.1.1 | 0.43 | 0.42 |
| South Korea | 2021-02-21 | 2021-06-13 | B.1.497 | 0.41 | 0.76 |

**Wave detection methodology.** Figure S4 A and B present the wave detection results in the United Kingdom when the incidence-based and change point detection methods are respectively applied (21, 22, 23, 24, 25). As mentioned in the paper, the incidence-based method misses important waves when a new wave starts meanwhile the incidence is still relatively high. On the other hand, the change-point detection method is very sensitive to short-term variations in the time series of cases. When both methods are combined as in the paper’s definition, they enable to have a more robust definition of infection waves, where every wave is associated with a new infectious variant in most cases (see section on the analysis of infection waves).


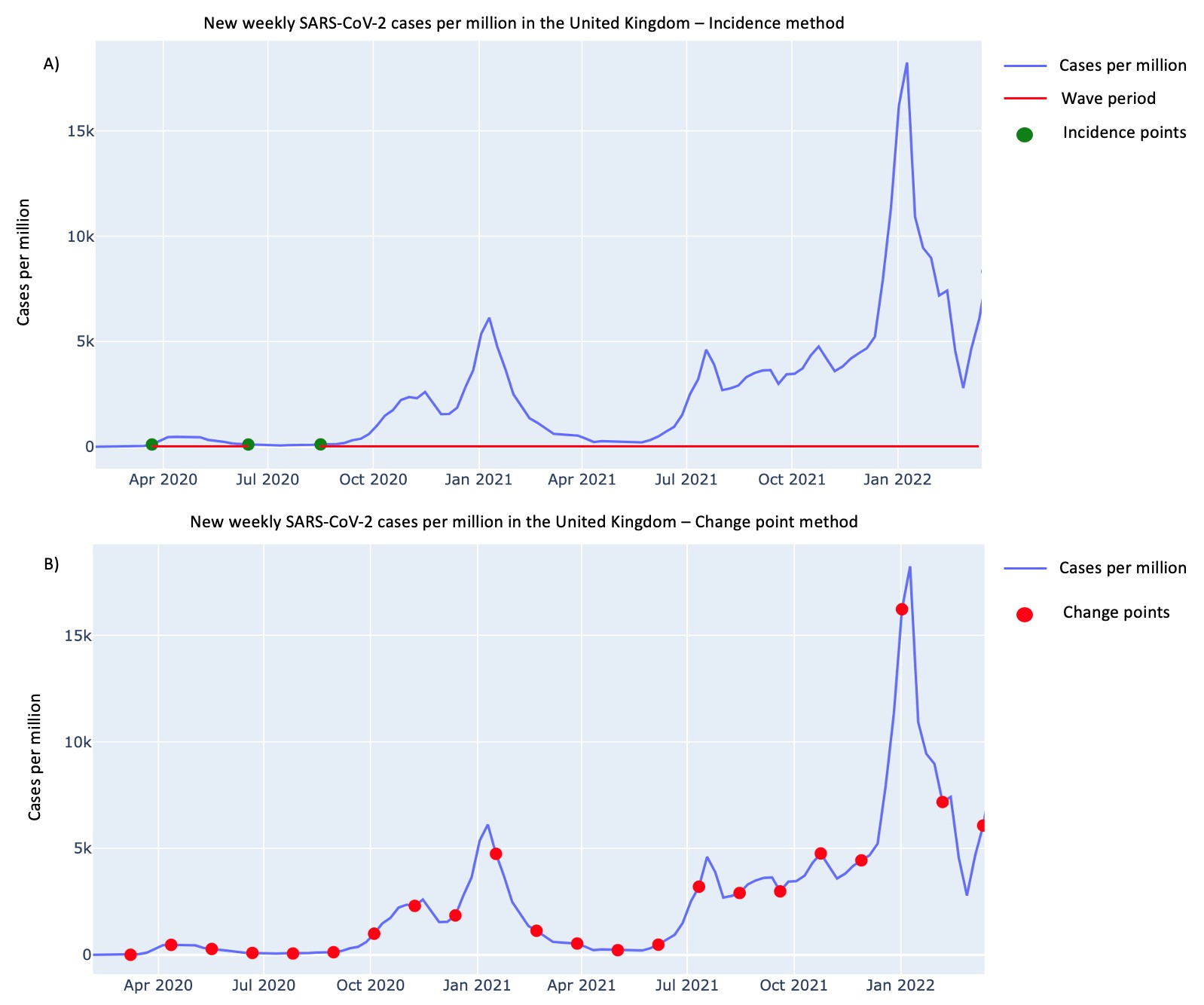


**Figure S4. Detection of SARS-CoV-2 infection waves in the United Kingdom using (A) the incidence-based method and (B) the change point detection method.**

**Table S3. Summary statistics on variants spread across countries**. It presents summary statistics for the top 20 most infectious variants in the dataset. It includes the number of countries where the variant has been detected, its maximum spread in cases per million, the number of countries where it caused more than 1000 cases per million (Above 1000), less than 1000 cases per million (Below 1000), and less than 500 cases per million (Below 500). Besides some exceptions, the table shows that although a variant can be infectious in a country, it could remain insignificant in the majority of the other countries, which implies that predictions of infectivity should be at the country level.

| Variant | Count countries | Maximum cases | Above 1000 | Below 1000 | Below 500 |
| --- | --- | --- | --- | --- | --- |
| BA.1.1 | 20 | 116264 | 15 | 5.0 | 4 |
| BA.1 | 26 | 110113 | 22 | 4.0 | 3 |
| B.1.1.7 | 30 | 30812 | 22 | 8.0 | 7 |
| B.1.177.60 | 6 | 29341 | 1 | 5.0 | 5 |
| AY.4.5 | 16 | 25378 | 1 | 15.0 | 14 |
| B.1.160 | 20 | 22113 | 13 | 7.0 | 5 |
| B.1.258.17 | 6 | 14752 | 1 | 5.0 | 4 |
| AY.4 | 26 | 14147 | 10 | 16.0 | 12 |
| Q.1 | 18 | 12642 | 1 | 17.0 | 17 |
| P.1 | 16 | 11723 | 1 | 15.0 | 14 |
| B.1.221 | 13 | 9933 | 7 | 6.0 | 5 |
| AY.122.1 | 4 | 9759 | 1 | 3.0 | 3 |
| B.1.177 | 22 | 8941 | 11 | 11.0 | 10 |
| AY.43 | 28 | 8373 | 16 | 12.0 | 9 |
| AY.127 | 20 | 7418 | 5 | 15.0 | 12 |
| AY.122 | 30 | 7194 | 7 | 23.0 | 17 |
| B.1.1.50 | 2 | 7190 | 1 | 1.0 | 1 |
| AY.45 | 11 | 6876 | 1 | 10.0 | 10 |
| B.1.362 | 3 | 6503 | 1 | 2.0 | 2 |
| P.2 | 5 | 6183 | 1 | 4.0 | 4 |

# Spread of variants over time and across countries in logarithmic scale. Figure S5 A plots the comparison of the number of cases caused by every variant 2 weeks and 3 months after its detection in logarithmic scale. Although the figure displays some level of correlation between the two, which is line with the results on the most predictive features of infectious variants, there is still a great level of variability in the 3-months spread for similar levels of spread after 2 weeks. Similarly, Figure S5 B compares the median spread to the maximum spread of across countries for every variant that has been infectious, i.e., causing more than 1000 cases per million, in at least one country. There also seems to be a correlation between the median and maximal spread. Note that that the value 6.9 in logarithmic scale corresponds to more than 1000 cases per million. Hence, this figure shows that for many variants with a high maximal spread, above 6.9, the median spread is low and below the equivalent threshold of infectious variants.

##
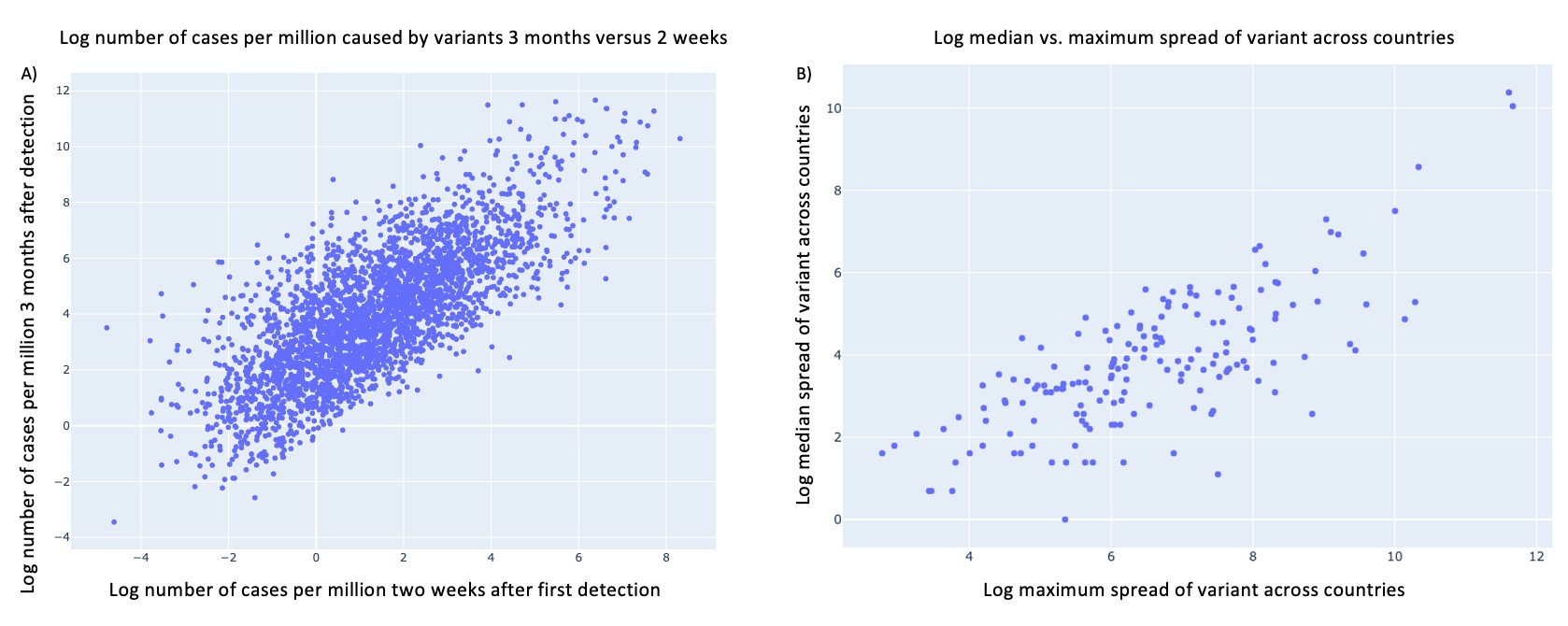


## Figure S5. Distribution of infection cases per variant and country (logarithmic scale). A) Comparison of the logarithm number of cases 2 weeks after first detection to 3 months afterwards for all the variants and countries under study. B) Comparison of the logarithm maximum and median spread across countries after 3 months of variants that were infectious in at least one country.

## Table S4. Features definition. It presents the features used as independent variables for the predictive models. They have been designed for every new variant in every country.

Feature Definition

| Mutations in Spike protein | Number of mutations in Spike protein |
| --- | --- |
| Mutations in N protein | Number of mutations in N protein |
| Mutations in M protein | Number of mutations in M protein |
| Mutations in E protein | Number of mutations in E protein |
| Mutations in NSP proteins | Number of mutations in NSP proteins |
| Ratio of 1*^st^* dominant variant | Ratio of sequences reported for 1*^st^* dominant variant among all other variants in the country during the observation period |
| Ratio of 2*^nd^* dominant variant | Ratio of sequences reported for 2*^nd^* dominant variant among all other variants in the country during the observation period |
| Week-distance | Jaccard-distance between the variant under study and the 1*^st^* dominant variant in the country during the observation period |
| Week-entropy | Entropy of the variants in the country during the observation period |
| Week-heterogeneity | Heterogeneity of the variants in the country during the observation period |
| Ratio among variants in week 0 | Ratio of sequences reported for the variant among all variants in the country during the week of detection |
| Ratio among variants in week 1 | Ratio of sequences reported for the variant among all variants in the country during the 1*^st^* week of observation |
| Ratio among variants in week 2 | Ratio of sequences reported for the variant among all variants in the country during the 2*^nd^* week of observation for the 2 weeks models |
| Cases of the variant in week 0 | Number of infections per million in the country for the variant during the week of detection |
| Cases of the variant in week 1 | Number of infections per million in the country for the variant during the 1*^st^* week of observation |
| Cases of the variant in week 2 | Number of infections per million in the country for the variant during the 2*^nd^* week of observation for the 2 weeks models |
| Maximum weekly cases of variant in other countries | Maximum weekly cases of the variant in other countries where it has been detected before or during the observation period |
| Total weekly cases | Total number of weekly cases in the country during the last week of the observation period |
| Percentage evolution of variant ratio week 1 | Percentage evolution of the ratio of the variant’s reported sequences in the country from week 0 to week 1 |
| Percentage evolution of variant ratio week 2 | Percentage evolution of the ratio of the variant’s reported sequences in the country from week 1 to week 2 for the 2 weeks models |
| Mean weekly percentage evolution of variant ratio | Mean weekly percentage evolution of the ratio of the variant’s reported sequences in the country during the observation period for the 2 weeks models |
| Mean change of variant ratio | Mean change of the variant’s ratio of reported sequences among all variants in the country from the week of detection to the last week of the observation period |
| Mean weekly second derivative of variant ratio | Mean weekly second derivative of the variant’s ratio of reported sequences in the country during the observation period |
| Percentage evolution of variant cases week 1 | Percentage evolution of the variant’s number of cases in the country from week 0 to week 1 |
| Percentage evolution of variant cases week 2 | Percentage evolution the variant’s number of cases in the country from week 1 to week 2 for the 2 weeks models |
| Mean weekly percentage evolution of variant cases | Mean weekly percentage evolution of the variant’s number of cases in the country during the observation period for the 2 weeks models |
| Mean change of variant cases | Variation of the variant’s number of cases in the country from the week of detection to the last week of the observation period |
| Mean weekly second derivative of variant cases | Mean weekly second derivative of the variant’s number of cases in the country during the observation period |
| Restrictions on gathering | 0 - No restrictions; 1 - Restrictions on very large gatherings, 2 gatherings between 100-1000 people, 3 gatherings between 10-100 people, 4 - gatherings of less than 10 people |
| Cancellation of public events | 0 - No measures; 1 - Recommended cancellations; 2 - Required cancellations |
| Vaccination rate | Rate of the total population vaccinated with at least one dose from any kind of SARS-CoV-2 vaccine by the last week of the observation period |

# Jaccard distance and phylogenetic distance. The traditional biology metric used to measure the evolution of a virus is the phylogenetic distance. This distance between two genetic sequences corresponds to the fraction of aligned positions in which the sequence has been changed, based on all the aligned nucleotides of the whole genome or a pre-selected gene or protein (30). However, the study adjusts this metric and uses the Jaccard distance instead that focuses on variations in the mutation sets of every variant. The following analysis justifies the choice of the paper to proceed with the Jaccard distance.

Table S5 shows a summary of phylogenetic and Jaccard distances between successive highly infectious variants. These results illustrate that successive SARS-CoV-2 infectious variants displayed barely detectable phylogenetic distance, meanwhile their Jaccard distance is relatively high.

## Table S5. Summary of Phylogenetic and Jaccard Distances

| Variant pairs | Phylogenetic distance | Jaccard distance |
| --- | --- | --- |
| B.1.1 - B.1.177 | 0.00094 | 0.80 |
| B.1.177 - B.1.1.7 | 0.0036 | 0.94 |
| B.1.1.7 - B.1.617.2 | 0.0082 | 0.97 |

Additionally, the study computes the week-distance, corresponding to the distance of each new variant from the dominant variant when it appears, based on the Jaccard distance and the phylogenetic distance. Subsequently, we will refer to these metrics by the week-Jaccard distance and the week-phylogenetic distance. The study then compares the distribution of these metrics for the group of variants in the training set that ended up being infectious, i.e., causing more than 1000 cases per million after 3 months, and those that ended up not being infectious. Then, a t-test is performed to compare the distribution of these metrics for infectious and non-infectious variants (41). The results show that the distributions of week-Jaccard distance enables to differentiate between the group of infectious and non-infectious variants (t-test = 2.97, p-value = 0.0029), whereas the week-phylogenetic distance distributions for infectious and non-infectious variants are not distinguishable (t-test = 1.4, p-value = 0.15). This result still holds when the phylogenetic distance is solely computed based on the spike protein (t-test = -0.06, p-value = 0.94). Hence, the Jaccard distance of every new variant from the dominant variant during its week of detection seems better suited to distinguish between the variants that will be infectious and those that will not. Moreover, the week-distance metric calculated with the phylogenic distance metric does not seem to have significant predictive power, nor does it improve the performance of the predictive models.

**Table S6. Out-of-sample predictive performance with 500 cases per million threshold.** As a robustness test, the study also tried 500 cases per million as a threshold for infectious variants. The results presented in Table S6 have a similar performance level to the main model.

| Observation period | AUC | Sensitivity | Specificity | Accuracy |
| --- | --- | --- | --- | --- |
| 1 week | 85.6% | 73.4% | 84.5% | 82.9% |
| 2 weeks | 88.0% | 79.9% | 83.1% | 82.7% |

**Table S7. Out-of-sample prediction and observed spread of AY.4 across countries**. It compares out-of-sample predictions for AY.4 after 2 weeks of observation to its actual spread after 3 months in every country. It shows that the model is able to have a differentiated prediction across countries. It identified all the countries where AY.4 has ultimately been infectious and those where it has not. The countries where the model made mistakes had less than 1000 cases per million of AY.4 but still a significant number of infections.

| Country | Cases per million after 3 months | Prediction above 1000 cases per million |
| --- | --- | --- |
| United Kingdom | 14147 | True |
| Ireland | 12104 | True |
| Slovenia | 4580 | True |
| Israel | 2096 | True |
| Lithuania | 1697 | True |
| Portugal | 1676 | True |
| Netherlands | 1362 | True |
| Switzerland | 1281 | True |
| Norway | 1098 | True |
| Denmark | 1020 | True |
| Spain | 883 | True |
| France | 779 | True |
| Belgium | 741 | True |
| Luxembourg | 542 | True |
| Sweden | 485 | True |
| Germany | 397 | False |
| Poland | 377 | True |
| Austria | 211 | True |
| Turkey | 210 | False |
| Finland | 159 | False |
| Australia | 149 | False |
| USA | 51 | False |
| Italy | 27 | False |
| Canada | 20 | False |
| Japan | 6 | False |
| India | 5 | False |

**Tables S8-S10.** Detection of infectious variants in sample countries. The tables below present the 5 most infectious variants, detected from April 2021 to March 2022, based on their respective reported infection cases after 3 months, in the United Kingdom, Israel, and South Africa. Their risk scores after 1 week of observation and 2 weeks of observation aiming to predict the variants which will cause more than 1000 cases per million are included in the tables. The risk score threshold for classifying variants as infectious is 0.14 after 1 week and 0.12 after 2 weeks of observation.

Table S8: Top 5 infectious variants in the UK detected from April 2021 to January 2022

| Pango lineage | Start of observation | Cases per million in week 1 | Cases per million in week 2 | Cases per million after 3 months | Risk score week 1 | Risk score week 2 |
| --- | --- | --- | --- | --- | --- | --- |
| BA.1 | 2021-11-21 | 12.67 | 83.15 | 53780.39 | 0.138 | 0.356 |
| BA.1.1 | 2021-11-28 | 8.47 | 65.46 | 28835.26 | 0.086 | 0.241 |
| BA.2 | 2021-12-26 | 664.31 | 77.3 | 24218.88 | 0.559 | 0.299 |
| AY.4 | 2021-04-25 | 8.22 | 27.05 | 14147.49 | 0.132 | 0.354 |
| AY.5 | 2021-04-25 | 2.07 | 4.37 | 922.94 | 0.057 | 0.014 |

Table S9: Top 5 infectious variants in Israel detected from April 2021 to January 2022

| Pango lineage | Start of observation | Cases per million in week 1 | Cases per million in week 2 | Cases per million after 3 months | Risk score week 1 | Risk score week 2 |
| --- | --- | --- | --- | --- | --- | --- |
| BA.1.1 | 2021-12-12 | 27.4 | 111.14 | 98380.91 | 0.428 | 0.690 |
| BA.1 | 2021-11-28 | 11.97 | 50.63 | 97803.77 | 0.241 | 0.765 |
| BA.2 | 2022-01-02 | 140.97 | 370.56 | 30115.79 | 0.436 | 0.267 |
| AY.122.1 | 2021-06-20 | 29.91 | 88.23 | 9758.79 | 0.356 | 0.741 |
| AY.122 | 2021-06-13 | 7.21 | 53.03 | 7194.39 | 0.347 | 0.698 |

Table S10: Top 5 infectious variants in South Africa detected from April 2021 to January 2022

| Pango lineage | Start of observation | Cases per million in week 1 | Cases per million in week 2 | Cases per million after 3 months | Risk score week 1 | Risk score week 2 |
| --- | --- | --- | --- | --- | --- | --- |
| BA.1 | 2021-11-14 | 52.02 | 189.5 | 8065.4 | 0.517 | 0.799 |
| AY.45 | 2021-05-16 | 12.79 | 16.09 | 6875.96 | 0.119 | 0.30 |
| BA.2 | 2021-12-05 | 57.49 | 105.66 | 2990.23 | 0.399 | 0.489 |
| AY.38 | 2021-05-30 | 30.22 | 72.45 | 1941.05 | 0.274 | 0.671 |
| AY.32 | 2021-05-30 | 13.43 | 26.34 | 1854.58 | 0.177 | 0.377 |
